# Hospital Festivals and Meetings

**Published:** 1894-07-28

**Authors:** 


### HOSPITAL FESTIVALS AND MEETINGS.

### DENTAL HOSPITAL OF LONDON.

A largely-attended conversazione in connection with the Dental Hospital of London was held at the Royal Institute Galleries, Prince's Hall, Piccadilly, on July 19th. Prizes were given during the evening to the successful students in the London School of Dental Surgery by Mr. Christopher Heath. Mr. Morton Smale, the Dean, took occasion to point out that the two chief prize-winners for the year—Mr. N. G. Bennett and Mr. U. S. Nowell—were both University men, a most satisfactory fact, and the first time he believed, that any students from either Oxford or Cambridge had entered the dental profession. Mr. Heath gave a short address after the presentation of prizes, in the course of which he reviewed the great progress made by the dental profession of late years. He could remember when it could be hardly said to exist. For the great alteration which had taken place much thanks were due to the present leaders of the profession, and especially to Sir John Tomes.

